# Evaluating a Washington DC Community-based meal-kit service aimed at mitigating dietary disparities: Results from the SouthEats pilot study

**DOI:** 10.1016/j.pmedr.2023.102382

**Published:** 2023-08-25

**Authors:** Joelle N. Robinson-Oghogho, Anne Palmer, Melissa Davey-Rothwell, Roland J. Thorpe Jr.

**Affiliations:** a Johns Hopkins Bloomberg School of Public Health, Department of Health Behavior and Society, Baltimore, MD, United States; b Johns Hopkins Center for Health Disparities Solutions, Baltimore, MD, United States; c Johns Hopkins Center for a Livable Future, Baltimore, MD, United States

**Keywords:** Meal-kits, Vegetables, Eating behaviors, Food access, Interventions, Pilot study, Cooking behaviors

## Abstract

**Background:**

Vegetable consumption is known to reduce the risk of various chronic health conditions. Yet a small percentage of US adults consume enough vegetables to meet national dietary guidelines. The SouthEats community-led meal–kit service was developed in Washington DC to address known barriers to healthy eating and vegetable consumption among middle- and low-income households.

**Methods:**

Using a series of online surveys, we conducted a pilot study to provide preliminary information on the influence of SouthEats on vegetable consumption and factors known to influence vegetable consumption. Wilcoxon matched-pair sign ranked tests were used to examine changes in key outcomes between baseline, midpoint and endpoint surveys.

**Results:**

Among SouthEats customers who completed all pilot study surveys (n = 23) there was a significant decrease in the median amount of time spent on meal prep between baseline and midpoint. Between baseline and endpoint, participants also indicated increased feelings that they had enough time to meet their needs including cooking healthfully. Overall vegetable and fruit and vegetable consumption increased between the baseline and midpoint but then decreased between the midpoint and endpoint survey periods. However, there were no statistically significant changes in the outcomes of home eating behaviors, perceptions of neighborhood healthy food access, vegetable self-efficacy, or vegetable consumption.

**Conclusion:**

Our results provide some preliminary evidence suggesting that the SouthEats meal-kit service could help reduce the amount of time spent on cooking, reduce feelings of time scarcity, and increase vegetable consumption in the short-term. Further research exploring this topic will require a larger study sample.

## Highlights

• Many factors contribute to dietary disparities among US urban populations.
• A novel meal-kit service reduces cooking time and may improve dietary patterns.
• Community-led interventions respond to people facing barriers to healthy eating.

## 1 Introduction

Although there is a robust understanding of the factors influencing vegetable consumption (Baranowski et al., 1999, Glanz et al., 1998), only 10% percent of US adults consume the recommended 2–3 cups of vegetables each day (Lee et al., 2022). Even greater disparities in vegetable consumption exist by race, income, gender, and geography, as shown by lower rates of consumption among people who are Black, male, have lower incomes, or live in rural or low-food access areas (Lee-Kwan et al., 2017, Hoy et al., 2017, Bahr, 2007, Chen et al., 2011, Rothgerber, 2013, O’Doherty Jensen and Holm, 1999, Kirkpatrick et al., 2012, Bowman, 2007, Vadiveloo et al., 2019, McCullough et al., 2022). Many interventions aim to increase vegetable consumption by educating consumers, increasing affordability through monetary supplements, or increasing accessibility by establishing healthy food retail outlets, but most of them fail to account for how known factors interact and compound health disparities. For example, since meals prepared at home are known to be more nutritious and more likely to contain vegetables than foods purchased for consumption away from home (Mancino et al., 2018, Todd et al., 2010, Fertig et al., 2019), many public health interventions explicitly and implicitly promote cooking at home and focus on enhancing opportunities to acquire raw vegetable products. However, many US adults can be characterized as being increasingly time-strapped (Beshara et al., 2010, Rogus, 2018).

Like other resources, time has been conceptualized as a health resource that is socially patterned and inequitably distributed across gender, income, and race (Gee et al., 2019, Strazdins et al., 2011). This is relevant in the case of food as federal food assistance programs prohibit the purchase of cooked foods and implement policies based on assumptions that recipients will spend an average of 16 h a week preparing meals; an amount of time higher than what the average working person spends on meal preparation (Jabs and Devine, 2006). As affordability, lack of time, employment status, and enjoyment have been identified as factors contributing to home cooking (Wolfson et al., 2016, Garcia et al., 2018) approaches to reduce diet-related disparities should account for time, place, and economic constraints on individuals’ access to healthy foods. Meal-kits are often marketed to people who want to cook from scratch but lack the time or skill to do so. These services simplify food prep by eliminating steps such as shopping for ingredients and planning menus. Meal-kits may also provide a useful introduction to cooking or increase efficacy around home food preparation behaviors.

To improve diet-related disparities, a group of Washington DC residents developed the SouthEats meal-kit service; supported through the Robert Wood Johnson Foundation Culture of Health Leaders program. By providing ready-to-cook meals, the SouthEats service attempts to address factors such as inequitable neighborhood access to healthy foods, affordability, individual cooking skills, and time constraints that limit home cooking. Like other meal-kit services, SouthEats provides customers with raw ingredients to prepare their choice of 3 meals each week. Customers also select their meal size (i.e., two servings vs. four servings per meal) based on their household needs and can choose to stop receiving meals as they wish. Unlike other meal-kits, SouthEats meals are pre-seasoned, cut/chopped, and pre-portioned, contain all necessary ingredients for preparation, require an average of 5 steps to prepare vs 15 steps in other meal-kit services, and cost $6.25 per serving compared to other leading meal-kit services with are priced around $9.99 per serving (SouthEats LLC, 2019, Statista, 2022). By providing meals that do not require prep-work, SouthEats targets customers who are particularly time-strapped or intimidated by cooking, while also allowing experienced cooks to integrate their own cooking practices. Additionally, Supplemental Nutrition Assistance Program (SNAP) participants were able to use their allotments to purchase the meals. Meals were ordered online and picked up at a farmers’ market centrally located in targeted neighborhoods (SouthEats LLC, 2019).

Little evidence exists on whether meal-kit products improve diet or vegetable consumption (Moores et al., 2021, Gibson and Partridge, 2019, Robinson-Oghogho et al., 2022). In recent years several community-based meal-kit interventions have been developed across the US targeting lower-income populations (Zeldman et al., 2020, Sweeney et al., 2021, Horning et al., 2021). Unlike existing research, this pilot study aims to evaluate the influence of the meal-kit service in a scenario where participants purchased the meal-kit service. Examining community-based social enterprises may provide additional insights on how to implement sustainable and tailored strategies to improve dietary behaviors among specific populations.

This pilot study was designed to provide preliminary information examining the influence of a community-led meal–kit service (SouthEats) on vegetable consumption, and factors known to influence vegetable consumption. Specifically, we assessed baseline characteristics and examined changes in vegetable consumption, home cooking behaviors, and perceptions among people in households receiving the SouthEats meal-kit service. We conceptualized self-efficacy for consuming vegetables, perceptions of neighborhood access to healthy foods, perceptions of time scarcity, time spent cooking, and frequency of cooking at home as anteceding outcomes to increased vegetable consumption. We hypothesized that utilization of the SouthEats meal-kits would increase vegetable consumption, vegetable consumption self-efficacy, perceived access to healthy foods, and the frequency of cooking at home; while decreasing perceptions of time scarcity, and time spent cooking.

## 2 Methods

### 2.1 Study recruitment and design

We conducted a prospective study using a series of online surveys to explore the potential of the SouthEats meal-kits as a tool for increased vegetable consumption among adults in low-and-middle-income households in Washington DC; defined as those with incomes ≤ 80% of the Area Median Income (i.e., ≤$74,837) (U.S. Census Bureau, 2021). Engagement and recruitment activities for both the SouthEats meal-kit service and the pilot study happened simultaneously through flyers distributed in three target neighborhoods; consisting of approximately 4,000 residential homes. The median household incomes for these neighborhoods ranged from $28,000 to $76,000, average household sizes were between 2 and 3 persons, and 94% to 95% of residents identified as Black or African American (United States Postal Service, 2021, U.S.Census Bureau, 2021).

Residents who purchased the SouthEats meals during the pilot period (i.e., July 26, 2021, through December 31, 2021) were invited and screened for study eligibility via email messages included with order confirmations. Eligible study participants were household members 18 years of age or older, who indicated they were responsible for at least 50% of the cooking or at least 50% of the food shopping in their household. Household membership was operationalized as sleeping and eating 4 or more nights a week at the residence. Adults who indicated their household received other commercial meal-kits were ineligible to participate in the study. SouthEats customers who indicated they were interested in joining the study were consented and enrolled. The 3-minute enrollment survey collected information on nine basic demographic questions and participant contact information. Participants were emailed baseline, midpoint, and endpoint surveys over eight weeks; with the baseline survey sent immediately after enrollment, the midpoint survey sent 30 days after completion of the baseline survey, and the endpoint survey sent 60 days after completion of the baseline survey. Each survey took approximately 25 min to complete. Participants received a $25 monetary incentive for each survey they completed. Participants who completed all three surveys received an additional $20 incentive. Data collection for this study was approved by the Johns Hopkins Bloomberg School of Public Health Institutional Review Board (IRB No: 14151).

### 2.2 Measures

#### 2.2.1 Key outcomes

The key outcome measures used to operationalize factors known to impact vegetable consumption were changes in at-home eating behaviors (Mancino et al., 2018, Todd et al., 2010, Fertig et al., 2019), time spent on food preparation (Pollard et al., 2002, Monsivais et al., 2014), perceived time scarcity (Rogus, 2018) perceived access to healthy foods (Erinosho et al., 2012, Caldwell et al., 2009), and self-efficacy for vegetable consumption (Campbell et al., 2008, Fernadez et al., 2015). At-home eating behaviors were assessed using two questions on how often participants ate foods prepared at home. Time spent on food preparation was assessed by asking participants to indicate the total number of hours and minutes spent cooking dinner. Perceived time scarcity was assessed using DeSousa et al. Perceived Time Scarcity subscale (DeSousa et al., 2020). Perceived neighborhood access to healthy foods was assessed using a six-item scale previously used by Ma et al. (Ma et al., 2016). Vegetable consumption self-efficacy was assessed using Mainvil et al., Self-efficacy to Eat Vegetables subscale (Mainvil et al., 2009).

Vegetable consumption was assessed using National Cancer Institute Fruit and Vegetable By-Meal screener (Thompson et al., 2002, National Cancer Institute, 2021). This instrument includes questions on the frequency and portion size of 9 food items (100% fruit juice, lettuce salad, French fries or fried potatoes, other white potatoes, beans, tomato sauce, vegetable soups, fruits, vegetables) with questions on fruit and vegetables asked for the morning, lunchtime/afternoon, and evening mealtimes. We report on total consumption of fruits and vegetables, total consumption excluding fruit juice and fruits, and total vegetables consumed during mealtimes, to provide estimates of the number of servings consumed daily.

#### 2.2.2 Participant characteristics

We also collected information on participant characteristics and baseline attitudes and behaviors. Baseline attitudes towards cooking were examined using the 6-item Convenience Orientation Scale (Candel, 2001) and 9 items were used to examine cooking aversion (Wolfson et al., 2016). On the Convenience Orientation Scale, higher scores indicated a stronger preference for cooking that is convenient and does not take much time to prepare. On the cooking aversion scale items expressing positive attitudes towards cooking were reverse coded and all 9 items were averaged to create a general cooking aversion score. Higher scores indicated a stronger dislike or aversion to cooking. We also reported on participant characteristics including age, race, educational attainment, diet-related health conditions, household income, household size, presence of children, grocery shopping frequency, and participation in any other local vegetable promotion programs or food access services. A detailed list of the questions used to collect key outcome and participant characteristic data is provided in Appendix A, Table A1.

### 2.3 Analysis

**Fig. 1:**
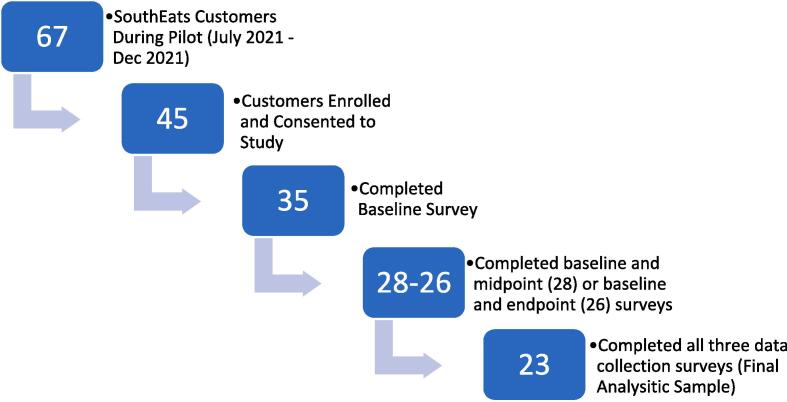
Description of 2021 SouthEats Pilot Study Recruitment and Participant Retention.

Of the 67 SouthEats customers who could have potentially joined the pilot study, 35 completed the baseline survey, 4 completed the baseline only, 5 completed the baseline and midpoint survey only, 3 completed the baseline and endpoint survey only, and 23 completed all three baseline, midpoint, and endpoint surveys. This is displayed in Fig. 1 below. Our final analytic sample includes 23 study participants who completed all three data collection surveys. To explore potential differences between participants in our analytic sample and those who enrolled but did not complete the study, we conducted bivariate analyses comparing the 23 participants who completed all three surveys, hereafter referred to as “completers,” to the 12 participants who only completed the baseline survey or the baseline and one other survey, hereafter referred to as “non-completers.” Given our small sample size, we used Fisher’s exact chi-square test to compare the two groups across various demographic and descriptive characteristics.

Among our analytic sample, we performed descriptive analyses of the primary outcomes of interest. We conducted the Shapiro Wilk test for normality to examine the distribution of the changes between time points for each outcome. Given our small sample size and non-normal distributions for several outcomes, we report proportions, median, and mean values. To examine changes, we conducted Wilcoxon matched-pair sign-ranked tests. This sign-ranked test is used to assess differences in paired observations when data are not normally distributed (MacFarland et al., 2016). We tested the null hypothesis that the distributions of each of our outcomes were the same in the baseline and follow-up surveys.

In this study, participants may have not received SouthEats meals at the time of taking the midpoint and endpoint surveys (e.g., choose to terminate service; did not order meals that week). To account for this, we also conducted sign rank tests among people who reported currently receiving meals from SouthEats at the respective midpoint and endpoint periods. All analyses were conducted in STATA.15 statistical software (StataCorp., 2017).

## 3 Results

Table 1 presents a descriptive summary of the study participants. The age of study participants ranged from 26 to 69 years old, with a mean age of 42. Over 85% of participants were Black (i.e., African American, Afro-Caribbean, African, or Black and another race). Seventeen percent of completer participants had household incomes of less than $25,000 (data not shown). Over one-third (35%) of completer participants had household incomes between $50,000 and $99,999, while 30% had incomes of $100,000 or more. Over half of completer participants lived in households with 3–5 people (52%), and households with children (57%). Sixty-one percent of completer participants indicated they were participating in food or other assistance programs. Completers differed from non-completers in their preference for cooking that is convenient and experiencing chronic health conditions, however, there were no significant differences between the two groups on any other demographic characteristic. Sixty-seven percent of non-completers (n = 8) compared to 35% completers (n = 8), reported having a chronic condition. This difference was significant at a 90% confidence level.

**Table 1:** Distribution of Characteristics Among 2021 SouthEats Pilot Study Participants.

		**Study Completer vs. Non-Completers**		
	**Total Participants n = 35**	**Non-Completers** **N = 12**	**Completers** **n = 23**	**p-value**
Age, mean(sd)	42.4 (11.4)	41.7 (9.9)	42.9 (12.3)	0.768
Race, %				1.000
Black Only & Black + Another	88.6	91.7	87.0	
White or Native	8.6	8.3	8.7	
Missing/No Response	2.9	0.0	4.4	
Chronic Health Condition, %				0.090
Any Condition	45.7	66.7	34.8	
No Condition	54.3	33.3	65.2	
Missing/No Response	0.0	0.0	0.0	
Educational Attainment, %				0.968
High School Grad or GED	5.7	0.0	8.7	
Some College or Tech School	22.9	25.0	21.7	
College Grad	31.4	33.3	30.4	
Grad School or Terminal Degree	37.1	41.7	34.8	
Missing/No Response	2.9	0.0	4.4	
Household Income, %				0.753
less than $49,999	22.9	16.7	26.1	
$50,000- - $99,999	37.1	41.7	34.8	
$100,000- $199,999	34.3	41.7	30.4	
Missing/No Response	5.7	0.0	8.7	
Household Size, %				0.758
1–2 members	40.0	50.0	34.8	
3–5 members	48.6	41.7	52.2	
6 or more members	8.6	8.3	8.7	
Missing/No Response	2.9	0.0	4.4	
Household Composition, %				0.736
Any Children	54.3	50.0	56.5	
Grocery Shopping Frequency, %				0.745
Once every two weeks	54.3	66.7	47.8	
Once a week	37.1	25.0	43.5	
More than once a week	8.6	8.3	8.7	
Missing/No Response	0.0	0.0	0.0	
Food Program or Assistance, %				0.318
Any Program	51.4	33.3	60.9	
No Programs	37.1	50.0	30.4	
Missing/No Response	11.4	16.7	8.7	
Cooking Preferences Score, mean^+^ (sd)	2.9 (1.1)	3.3 (1.5)	2.7(0.8)	0.145
Cooking Convenience Score, mean^++^ (sd)	4.7(0.99)	5.1 (1.2)	4.4 (0.82)	**0.061**

p-values indicate if a statistically significant difference exists between participants who completed the baseline survey but did not complete subsequent surveys (n = 12) and those who completed all three surveys (n = 23), using paired *t*-test for continuous variables and Fishers exact chi-squared test for categorical variables, at a 95% confidence level.

+ The highest possible score for cooking aversion is seven. Higher scores indicate a stronger dislike or aversion to cooking.

++ The highest possible score for cooking convenience is seven. Higher scores indicate a stronger preference for cooking that is convenient and does not take much time.

Regarding participants’ attitudes towards cooking, the maximum score for the cooking aversion and convenience scales was 7, with a neutral score of 3.5. Among all participants who completed the baseline survey, the average score for cooking aversion was 2.92, while the average cooking convenience score was 4.65., suggesting participants had a slightly elevated proclivity for convenient or fast cooking and a slight preference or enjoyment of cooking. Completers and non-completers had similar proclivities or enjoyment of cooking (2.74 vs. 3.34, p = 0.145). However, cooking convenience scores were slightly lower among completer participants (4.43 vs. 5.08, p = 0.061), suggesting that those who did not complete all three surveys had a higher preference for cooking that is convenient than people who completed all the follow-up surveys.

The mean and median values for study outcomes at baseline, midpoint, and endpoint among completer participants are provided in Table 2. Overall, the median number of times study participants cooked dinner increased from 4 to 5 times a week, and the number of minutes spent cooking decreased. Measures assessing participants’ perceptions of their time availability, neighborhood access to healthy foods, and self-efficacy for vegetable consumption were only asked at baseline and endpoint. Overall, the mean and median values for perceived time scarcity, food access, and vegetable self-efficacy increased slightly from baseline to endpoint time periods. Measures used to access vegetable consumption showed an increase in the median number of cups of fruit and vegetables consumed between baseline and midpoint but a decrease between midpoint and endpoint in total fruit and vegetable consumption.

**Table 2:** Distribution of 2021 SouthEats Pilot Study Outcomes at Baseline, Midpoint, and Endpoint Among Participants Completing All Three Data Collection Surveys n = 23.

	**Baseline**		**Midpoint**		**Endpoint**	
**INFLUENCING FACTORS**						
**Meals Eaten Out**	**n (%)**		**n (%)**		**n (%)**	
1–3 meals	14 (60.9)		16 (69.6)		14 (60.9)	
4–7 meals	4 (17.4)		5 (21.7)		8 (34.8)	
8–10 meals	4 (17.4)		1 (4.4)		1 (4.4)	
11–14 meals	0 (0.0)		1 (4.4)		0 (0.0)	
Missing/No Response	1 (4.4)		0 (0.0)		0 (0.0)	
	**mean** **(sd)**	**median** **(IQR)**	**mean** **(sd)**	**median** **(IQR)**	**mean** **(sd)**	**median** **(IQR)**
**Cooking Frequency**	4.25 (1.73)	4 (3,5)	4.77 (2.51)	5 (3,6)	3.77 (1.45)	4 (3,5)
**Minutes Cooking**	270 (2 9 0)	150 (105,360)	167 (1 5 7)	120 (60,180)	194 (2 5 4)	120 (60,240)
**Enough Time to Cook Healthy**^a^	3.35 (1.19)	3 (2,4)	–		4.55 (1.18)	5 (4,6)
**Time Scarcity Score**^b^	3.16 (0.76)	3.13 (2.5,3.88)	–		3.89 (0.95)	4 (3.69,4.25)
**Perceived Food Access Score**^c^	2.37 (0.77)	2.19 (1.88,2.63)	–		2.58 (0.73)	2.38 (2.13,3.63)
**Vegetable Self Efficacy Score**^d^	4.12 (0.93)	4.43 (3.57,5)	–		4.23 (0.77)	4.36 (3.5,4.93)
**VEGETABLE CONSUMPTION**						
**Cups of Vegetables Consumed During Meals**	1.17 (1.45)	0.70 (0.21,1.66)	1.03 (0.83)	0.88 (0.43,1.5)	0.80 (0.79)	0.59 (0.29,0.91)
**Cups of Foods Except Fruits**	2.15 (1.98)	1.43 (0.91,2.73)	1.95 (1.30)	1.72 (0.90,3.18)	1.63 (1.53)	1.24 (0.75,1.77)
**Cups of Fruits and Vegetables**	3.14 (2.59)	1.90 (1.27,4.60)	2.97 (2.19)	2.40 (1.55,3.88)	2.68 (2.05)	2.04 (1.37,4.07)

Baseline survey was administered at enrollment/purchase.

Midpoint survey was administered 30 days after completion of the baseline survey (i.e., 4 weeks).

Endpoint survey was administered 60 days after completion of the baseline survey (i.e., 8 weeks.

a Having enough time to cook was assessed using a 7-point Likert scale of strongly disagree to strongly agree. Higher scores indicate affirmation of having enough time to cook healthy meals.

b The highest possible time scarcity score is 7. A higher score indicates less time scarcity.

c The highest possible perceived food access score is 5. A higher score indicates perceptions of having greater access to healthy food.

d The highest possible vegetable self-efficacy score is 5. A higher score indicates greater confidence in the ability to eat vegetables.

Table 3 presents the median changes in study outcomes between each data collection period, among completer participants and among completers who also reported receiving meals from SouthEats at the time of the survey. Between baseline and endpoint, participants reported statistically significant increases in feeling they had enough time to cook a healthy meal, and less time scarcity. When examining changes in the reported amount of time spent cooking, participants reported a median decrease of 60 min (IQR: −180, 15) between baseline and midpoint surveys, which was found to be statically significant. There were no statistically significant changes in at home eating behaviors (i.e., number of meals eaten out or cooking frequency), perceptions of neighborhood access to healthy food, or vegetable self-efficacy. The results among the subpopulation who reported receiving meals at the time of the endpoint surveys were consistent with what is reported among the overall completer group, albeit larger.

**Table 3:** Changes in Study Outcomes between Baseline, Midpoint, and Endpoint Among 2021 SouthEats Pilot Study Participants Completing All Three Data Collection Surveys n = 23.

	**Changes Baseline to Midpoint**		**Changes Midpoint to Endpoint**		**Changes Baseline to Endpoint**	
	Median Difference (IQR)	p-value	Median Difference (IQR)	p -value	Median Difference (IQR)	p -value
**INFLUENCING FACTORS**						
**Meals Eaten Out**						
All Completers	0 (0, 0)	0.685	0 (0, 0)	1.00	0 (0, 0)	0.879
Completer w. Meals at Time Point	0 (-1, 0)	0.665	0 (0, 0)	1.00	0 (0, 0)	0.298
**Cooking Frequency**						
All Completers	0 (-1, 3)	0.621	0 (-3, 1)	0.206	−1 (-2, 1)	0.430
Completer w. Meals at Time Point	0 (-1, 3)	0.401	0 (0, 2)	0.663	0.5 (-1, 2)	0.782
**Minutes Cooking**						
All Completers	−60 (-180, −15)	**0.007**	0 (-135, 45)	0.927	−60 (-180, 15)	0.088
Completer w. Meals at Time Point	−60 (-150, −15)	**0.011**	7.5 (-15, 195)	0.257	−44.8 (-75, 60)	0.615
**Enough Time to Cook Healthy**						
All Completers	–	–	–	–	1 (1,1)	**0.001**
Completer w. Meals at Time Point	–	–	–	–	1 (1,1)	**0.004**
**Time Scarcity Score**						
All Completers	–	–	–	–	0.625 (0.13, 1.31)	**0.001**
Completer w. Meals at Time Point	–	–	–	–	0.563 (0.06, 1.13)	**0.031**
**Perceived Food Access Score**						
All Completers	–	–	–	–	0.125 (-0.13, 0.63)	0.287
Completer w. Meals at Time Point	–	–	–	–	0.125 (-0.13, 0.63)	0.597
**Vegetable Efficacy Score**						
All Completers	–	–	–	–	0 (-0.14, 0.57)	0.644
Completer w. Meals at Time Point	–	–	–	–	0.143 (-0.14, 0.57)	0.211
**VEGETABLE CONSUMPTION**						
**Cups of Vegetables During Meals**						
All Completers	0.125 (-0.25, 0.48)	0.402	-0.25 (-0.5, 0.05)	**0.032**	0 (-0.43, 0.38)	0.681
Completer w. Meals at Time Point	0.215 (0.0, 0.48)	0.097	-0.348 (-0.5, -0.05)	**0.009**	0.063 (-0.21, 0.46)	0.530
**Cups of Foods Except Fruits**						
All Completers	0.075 (-1.29, 0.96)	0.879	-0.30 (-0.82, 0.18)	0.136	0.178 (-0.37, 0.54)	0.952
Completer w. Meals at Time Point	0.178 (-0.57, 0.96)	0.332	-0.177 (-0.54, 0.18)	0.397	0.215 (-0.36, 0.63)	0.551
**Cups of Fruits and Vegetables**						
All Completers	0.018 (-0.93, 0.95)	0.927	-0.111 (-1.00, 0.68)	0.648	0.293 (-0.61, 0.92)	0.831
Completer w. Meals at Time Point	0.533 (-0.72, 0.96)	0.356	0.007 (-0.65, 0.64)	0.975	0.379 (-0.42, 1.07)	0.551

p-values indicate if a significant difference exists in the median value of the outcomes between the specified timepoints (i.e., baseline to midpoint, midpoint to endpoint, or baseline to endpoint), at a 95% confidence level using the Wilcoxon matched-pair sign rank test.

The baseline to midpoint time period was 30 days after completion of the baseline survey (i.e., 4 weeks).

The midpoint to endpoint time period was 60 days after completion of the baseline survey (i.e., 8 weeks).

**Fig. 2:**
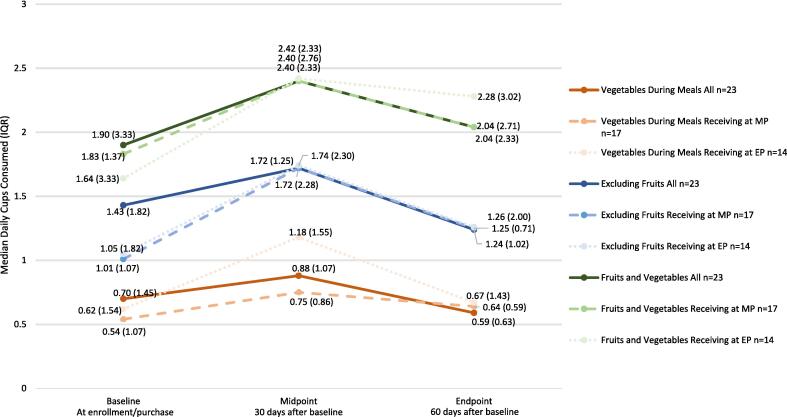
Median Estimated Daily Fruit and Vegetable Consumption Among 2021 SouthEats Study Participants Completing All Three Data Collection Surveys and Who Reported Receiving SouthEats Meals at Midpoint or Endpoint.

There were no statistically significant changes in vegetable consumption, between baseline and midpoint among completer participants. However participants who reported continuing to receive meals from SouthEats at midpoint increased vegetable consumption by 0.215 (IQR: 0.0 to 0.48) cups. Overall, vegetable consumption decreased between midpoint and endpoint time periods. As depicted in Fig. 2, participants who reported continuing to receive SouthEats meals at the time of the endpoint survey showed the largest increases in vegetable consumption between baseline and midpoint, and baseline and endpoint, and the smallest decreases in consumption between midpoint and baseline.

## 4 Discussion

The goal of this study was to provide pilot data examining the influence of the SouthEats meal-kit service on vegetable consumption and factors known to influence vegetable consumption among low- and middle-income adults in the Washington DC region. Many of the study outcome values moved in the hypothesized direction between the baseline and midpoint time periods, but generally dropped off between the midpoint and endpoint. Our results, including the observed attenuation, are consistent with other similar studies (Horning et al., 2021, Wark et al., 2018), and provide preliminary evidence suggesting that the SouthEats meal-kit service could help reduce the time spent on cooking, reduce feelings of time scarcity, and increase vegetable consumption in the short-term.

The SouthEats meal-kit business was developed in response to the inequitable healthy food landscape in Washington DC, which is also seen in many US urban areas. Additionally, the service was launched during the COVID-19 pandemic (i.e., July – December 2021), which left many people sequestered in their homes, and likely compounded the situation of those already experiencing inequitable access to food. It is likely that SouthEats, like many other meal-kit services, experienced increased interest during the pandemic that may have dampened as the COVID-19 vaccine became widely accessible and restrictions began to be lifted (Tolbert et al., 2022, Centers for Disease Control and Prevention, 2022). Nonetheless, as SouthEats and other meal-kit services continue to operate they may present an opportunity to improve health disparities if they are developed with the people who experience the greatest burdens in mind.

As meal-kit users have traditionally been characterized as younger and high-income earners, there has been a paucity of scientific research examining the implications of meal-kits on nutritional outcomes, specifically among low- and middle-income adults. Our study attempted to fill this gap by exploring diet-related outcomes among this population. Sixty-one percent of participants in our analytic sample reported household incomes less than $99,000, and 39% indicated they were SNAP participants. Although the household incomes of our study participants were higher than national averages, they were still generally lower than the median household income for Washington DC (U.S. Census Bureau, 2021). Coupled with the fact that over 90% of our participants resided in zip codes with large areas identified as low-income and low-food access areas (U.S. Department of Agriculture (USDA), 2022) our study population generally represents the target population of low- and middle-income adults in low food access areas in Washington DC.

One notable finding from our analyses was the influence of the meal-kit services on time spent preparing food and perceived time scarcity among participants. Between baseline and midpoint, people who received meals from SouthEats spent significantly less time cooking and felt they had enough time to prepare healthy meals. On average, Americans spend 85 min on food preparation and clean-up (Anekwe and Zeballos, 2019). In our pilot study, participants initially reported spending even more time on food preparation activities. Although increased time spent on food preparation and cooking has been associated with greater consumption of vegetables and fruits (Monsivais et al., 2014), if food preparation time could be reduced in a way that does not diminish the nutritional or hedonic integrity of the foods consumed, as with SouthEats, similar services could be a viable option to increase at home eating and improve nutritional outcomes. Since time could be considered a diminished health resource among lower-income, working, Black and Latin women (Gee et al., 2019), services such as SouthEats that are created to address the challenges specifically faced by these populations may provide increased potential for improving dietary outcomes.

This study is not without limitations. First, the final sample used in these analyses was notably small, with just 23 participants. This severely limited the types of statistical analyses we were able to conduct. Participants in our analytic sample purchased SouthEats meals for a median of 6 weeks (IQR: 4, 8.75). Although our analyses segmented users who reported continuing to receive the meals at the time of the survey, it is possible that some users did not choose to continue receiving the meals long enough to experience changes. However, the goal of the study was to gather pilot data to explore the potential of the SouthEats initiative on vegetable consumption and factors associated with vegetable consumption. Our findings suggest that future similarly designed studies aiming to test the impact of SouthEats or other similar services on vegetable consumption should aim to recruit a study sample with enough power to detect changes of no more than 0.20 cups. As noted above, during the time of this pilot study SouthEats only acquired 67 customers. The company had to adjust its community engagement activities which likely reduced the number of people they were able to attract because of the COVID-19 pandemic.

Despite these limitations, this study possesses several strengths. Since perceived and material barriers to accessing vegetables have been identified as significant factors in predicting adult vegetable consumption behaviors, this analysis attempted to examine both structural, material, and psychosocial influences on vegetable consumption. Additionally, participants purchased the meals rather than receiving them for free, as is often the case in many pilot interventions. This feature not only reduced food waste, but also allowed for the examination of the feasibility of the service in a real-world context. Further, the inclusion of participants who used their SNAP benefits to purchase SouthEats meals is another strength. Although SNAP allocations are known to be insufficient to meet the nutritional needs of most households (Figueroa, 2020), this analysis provides some preliminary data to suggest that meal-kits may help promote vegetable consumption in this population as well.

## 5 Conclusion

The examination of a community-led meal-kit service to explore its potential as a tool for bolstering vegetable consumption among adults in low-and-middle-income households is an area of research with the potential to help narrow diet-related disparities. Our findings may add to the body of evidence on approaches to increasing vegetable consumption, specifically community-based approaches that are designed by members of the target audience. The information gained from this analysis provides foundational insight on how to improve vegetable consumption in a real-world context among lower income people, although a larger study sample would be required to examine this relationship more rigorously. By adding to the body of evidence about meal services that increase vegetable consumption, we may inform services included in local and national supplemental food programs or scaled for community level impacts.

## Funding

This work was supported by the Johns Hopkins University Bloomberg American Health Initiative (Grant Number: 1600108315 and 1600108350). The funding entities had no role in the study design, analysis, or writing of this publication.

## CRediT authorship contribution statement

**Joelle N. Robinson-Oghogho:** Data curation, Conceptualization, Methodology, Formal analysis, Writing – original draft. **Anne Palmer:** Writing – review & editing. **Melissa Davey-Rothwell:** Conceptualization, Writing – review & editing. **Roland J. Thorpe Jr.:** Methodology, Writing – review & editing, Supervision.

## Declaration of Competing Interest

The authors declare the following financial interests/personal relationships which may be considered as potential competing interests: Joelle Robinson-Oghogho is a co-founder and worker-owner member of the SouthEats community-based meal-kit service. The remaining authors declare that the research was conducted in the absence of any commercial or financial relationships that could be construed as a potential conflict of interest.

## Figures and Tables

**Table A1:** Detailed Description of SouthEats Pilot Study Measures.

**Variable**	**Survey Questions and Measures**
**Key Outcomes**	
changes in at-home eating behaviors	“During the past 7 days, how many meals did you get that were prepared away from home in places such as restaurants, fast food places, food stands, grocery stores, or from vending machines? Thinking about breakfast, lunch, or dinner.” Categorical response options were “1-3, 4-7, 8-10, or 11-14.” “In the past 7 days, how many times did you or someone in your household cook dinner?”
time spent on food preparation	“During the past 7 days, how much time did it usually take you to cook dinner? Think about how many hours on average you spend preparing, cooking, and cleaning up from meals each time.”
perceived time scarcity scale	We asked participants to rate their level of agreement with the statements “I have enough time to exercise,” “I have enough time to engage in hobbies or engage in activities I enjoy,” “I have more to do than I have time to do it in,” “I have enough time to meet all of my responsibilities,” “Even though I am able to get done what needs to get done, I often feel like I do not have enough time,” “I have enough time to get done what needs to get done for work/school,” “I have enough time to cook healthy meals,” “I have enough time to spend with family/friends” Items were scored in accordance with the scale developer.
perceived access to healthy foods scale	We asked participants to rate their level of agreement with the statements “How much of a problem would you say that lack of access to adequate food shopping is in your neighborhood?,” “A large selection of fresh fruits and vegetables is available in my neighborhood,” “The fresh fruits and vegetables in my neighborhood are of high quality,” “The fresh fruits and vegetables in my neighborhood are affordable,” “A large selection of low-fat products is available in my neighborhood,” “There are many opportunities to purchase fast foods in my neighborhood such as McDonald’s, Taco Bell, KFC and takeout pizza places, etc.,” “In my neighborhood, it is easy and convenient to buy healthy foods,” and “I am usually able to get the kinds of foods that I want to eat.” Items were scored in accordance with the scale developer.
self-efficacy for vegetable consumption	We asked participants to rate their level of confidence with the statements “I can eat vegetables even when I have to prepare them myself,” “I can eat at least two different vegetables during my main meal on most days” “I can eat vegetables even on days when I am in a rush,” “I can eat vegetables when I am tired and have to prepare them”Measured using a 5-point Likert scale (1 = Not at all confident 5 = Totally confident)
**Participant Characteristics**	
grocery shopping frequency	“How often do you go grocery shopping?” Response options “once a month,” “once every two weeks,” “once a week,” and “more than once a week.”
cooking convenience	We asked participants to rate their level of agreement with the statements “The less physical energy I need to prepare a meal, the better,” “The ideal meal can be prepared with little effort,” “I prefer to spend as little time as possible on meal preparation,” “I want to spend as little time as possible cooking,” “At home I prefer to eat meals that can be prepared quickly,” and “It's a waste of time to spend a long time in the kitchen preparing a meal.” Measured using a 7-point Likert scale (1= Strongly Disagree 7 = Strongly Agree).
cooking aversion	We asked participants to rate their level of agreement with the statements “I enjoy cooking,” “cooking takes too much time,” “cooking cost too much money,” “cooking meals at home helps me eat more healthfully,” “I am a good cook,” “cooking is important to me,” “cooking is a burden or chore,” “cooking is stressful,” and “cooking for others makes me feel happy.”7-point Likert scale (1= Strongly Disagree 7 = Strongly Agree). Items expressing positive attitudes towards cooking were reverse coded and all 9-items were averaged to create a general cooking aversion score.
age	What is your month and year of birth?
race	Which one of these groups would you say best represents your race and ethnicity? (check all that apply)Response options were “African American,” “Afro Caribbean or African,” “Hispanic,” “White,” “East Asian,” “South Asian or Pacific Islander,” “Native American/First Nation,” and “Other”Race was categorized into two groups Black—which included people who identified as African American; Afro-Caribbean or African; African American and East Indian; or African American, White, and Native American—and White or Native American –which included people who identified as White; White and Native American; or Native American
educational attainment	What is the highest level or year of school you completed?” Responses were categorized into four groups “High school graduate or GED (Grade 12), Some College or Technical School, College Graduate, and Graduate School or Terminal Degree.”
diet-related health conditions	“Has a doctor, nurse, or other health professional ever told you that you had any of the following….hypertension also called high blood pressure, coronary heart disease, heart attack also called myocardial infarction, diabetes, pre-diabetes or borderline diabetes, stroke, or kidney disease (not including kidney stones, bladder infection or incontinence).” We categorized participants as having a diet-related chronic health condition if they answered “Yes” for any condition in response to the questions
household income	“Which of the following best describes your household income before taxes?” Participants were able to select from 11 household income categories ranging from “less than $10,000 to $200,000 or more.” Responses were categorized into three groups “less than $49,999, $50,000- $99,999, and $100,000- $199,999.
household size and presence of children in the household	Household size and household compositions were assessed through one question that ask participants to indicate how many people including themselves lived in their household that were less than 5 years, 5-17 years old, 16-65 years old, and 65 years and older.
participation in other local vegetable promotion/ food access programs	“Did you or anyone in your household receive benefits from any of the following programs in the past 30 days? (check all that apply)” Response options were “SNAP/EBT, WIC (Women Infants and Children), TANF, Grocery Plus/Commodity Supplemental Food Program, Food Pantry/Capital Area Food Bank, Produce Plus/Farmers Market Bonus Bucks, Home Delivered Prepared Meals (i.e. Meals on Wheels/ Food and Friends), Home Delivered Groceries (i.e. Pea Pod, Amazon Fresh, Food and Friends), Medical Meals (i.e. AmeriHealth Caritas DC), Produce Prescription/ Produce Rx or Produce Plus, Commercial Weight Loss Programs (Jenny Craig, Nutri-System, Other, and Have not participated in any of these programs” We created a binary variable for participation in any program or not participating in any program or service.

## Data Availability

Data will be made available on request.

## Appendix A

Table A1.

## References

[b0005] Anekwe TD, Zeballos E. Food-Related Time Use: Changes and Demographic Differences. United States Department of Agriculture, Economic Research Service; 2019.

[b0010] Bahr P.R. (2007). Race and nutrition: an investigation of Black-White differences in health-related nutritional behaviours. Sociol. Health Illn..

[b0015] Baranowski T., Cullen K.W., Baranowski J. (1999). Psychosocial correlates of dietary intake: Advancing dietary intervention. Annu. Rev. Nutr..

[b0020] Beshara M., Hutchinson A., Wilson C. (2010). Preparing meals under time stress. The experience of working mothers. Appetite.

[b0025] Bowman S. (2007). Low economic status is associated with suboptimal intakes of nutritious foods by adults in the National Health and Nutrition Examination Survey 1999–2002. Nutr. Res..

[b0030] Caldwell E.M., Miller Kobayashi M., DuBow W.M., Wytinck S.M. (2009). Perceived access to fruits and vegetables associated with increased consumption. Public Health Nutr..

[b0035] Campbell M.K., McLerran D., Turner-McGrievy G., Feng Z., Havas S., Sorensen G., Buller D., Beresford S.A.A., Nebeling L. (2008). Mediation of adult fruit and vegetable consumption in the national 5 a day for better health community studies. Ann. Behav. Med..

[b0040] Candel M.J.J.M. (2001). Consumers' convenience orientation towards meal preparation: conceptualization and measurement. Appetite.

[b0045] Centers for Disease Control and Prevention. COVID Data Tracker Atlanta, GA: US Department of Health and Human Services, CDC; 2022 [March 18, 2022:[Available from: https://covid.cdc.gov/covid-data-tracker.

[b0050] Chen X.L., Cheskin L.J., Shi L.Y., Wang Y.F. (2011). Americans with Diet-Related Chronic Diseases Report Higher Diet Quality Than Those without These Diseases. J. Nutr..

[b0055] DeSousa M., Reeve C.L., Peterman A.H. (2020). Development and initial validation of the Perceived Scarcity Scale. Stress. Health.

[b0060] Erinosho T.O., Oh A.Y., Moser R.P., Davis K.L., Nebeling L.C., Yaroch A.L. (2012). Association Between Perceived Food Environment and Self-Efficacy for Fruit and Vegetable Consumption Among US Adults, 2007. Prev. Chronic Dis..

[b0065] Fernadez B.R., Warner L.M., Knoll N., Montenegro E.M., Schwarzer R. (2015). Synergistic effects of social support and self-efficacy on dietary motivation predicting fruit and vegetable intake. Appetite.

[b0070] Fertig A.R., Loth K.A., Trofholz A.C., Tate A.D., Miner M., Neumark-Sztainer D., Berge J.M. (2019). Compared to Pre-prepared Meals, Fully and Partly Home-Cooked Meals in Diverse Families with Young Children Are More Likely to Include Nutritious Ingredients. Journal of the Academy of. Nutr. Diet..

[b0075] Figueroa E, Legendre J. States That Still Impose Sales Taxes on Groceries Should Consider Reducing or Eliminating Them2020 June 10, 2022. Available from: https://www.cbpp.org/research/state-budget-and-tax/states-that-still-impose-sales-taxes-on-groceries-should-consider#_ftn7.

[b0080] Garcia M.T., Sato P.M., Trude A.C.B., Eckmann T., Steeves E.T.A., Hurley K.M., Bógus C.M., Gittelsohn J. (2018). Factors Associated with Home Meal Preparation and Fast-Food Sources Use among Low-Income Urban African American Adults. Ecol. Food Nutr..

[b0085] Gee G.C., Hing A., Mohammed S., Tabor D.C., Williams D.R. (2019). Racism and the Life Course: Taking Time Seriously. Am. J. Public Health.

[b0090] Gibson A.A., Partridge S.R. (2019). Nutritional Qualities of Commercial Meal Kit Subscription Services in Australia. Nutrients.

[b0095] Glanz K., Basil M., Maibach E., Goldberg J., Snyder D. (1998). Why Americans eat what they do: Taste, nutrition, cost, convenience, and weight control concerns as influences on food consumption. J. Am. Dietetic Assoc..

[b0100] Horning ML, Hill T, Martin CL, Hassan A, Petrovskis A, Bohen L. The East Side Table Make-at-Home Meal-Kit Program is feasible and acceptable: A pilot study. Appetite. 2021;160. 10.1016/j.appet.2020.105087 PMC7878310 33359465

[b0105] Hoy M.K., Goldman J.D., Moshfegh A.J. (2017). Differences in fruit and vegetable intake of U.S. adults by sociodemographic characteristics evaluated by two methods. J. Food Compos. Anal..

[b0110] Jabs J., Devine C.M. (2006). Time scarcity and food choices: An overview. Appetite.

[b0115] Kirkpatrick S.I., Dodd K.W., Reedy J., Krebs-Smith S.M. (2012). Income and Race/Ethnicity Are Associated with Adherence to Food-Based Dietary Guidance among US Adults and Children. J. Acad. Nutr. Diet..

[b0120] Lee SH, Moore LV, Park S, Harris DM, Blanck HM. Adults Meeting Fruit and Vegetable Intake Recommendations - United States, 2019. MMWR Morb Mortal Wkly Rep. 2022;71(1):1-9. doi: http://dx.doi.org/10.15585/mmwr.mm7101a1. 10.15585/mmwr.mm7101a1 PMC8735562 34990439

[b0125] Lee-Kwan SH, Moore LV, Blanck HM, Harris DM, Galuska D. Disparities in State-Specific Adult Fruit and Vegetable Consumption - United States, 2015. Mmwr-Morbidity and Mortality Weekly Report. 2017;66(45):1241-7. doi:http://dx.doi.org/10.15585/mmwr.mm6645a1. 10.15585/mmwr.mm6645a1 PMC5726245 29145355

[b0130] Ma X., Liese A.D., Bell B.A., Martini L., Hibbert J., Draper C., Burke M.P., Jones S.J. (2016). Perceived and geographic food access and food security status among households with children. Public Health Nutr..

[b0135] MacFarland T.W., Yates J.M., MacFarland T.W., Yates J.M. (2016). IntRoduction to NonpaRametRic Statistics foR the Biological Sciences Using R.

[b0140] Mainvil L.A., Lawson R., Horwath C.C., McKenzie J.E., Reeder A.I. (2009). Validated Scales to Assess Adult Self-Efficacy to Eat Fruits and Vegetables. Am. J. Health Promot..

[b0145] Mancino L, Guthrie J, Ver Ploeg M, Lin B-H. Nutritional Quality of Foods Acquired by Americans: Findings From USDA's National Household Food Acquisition and Purchase Survey 2018.

[b0150] McCullough M.L., Chantaprasopsuk S., Islami F., Rees-Punia E., Um C.Y., Wang Y., Leach C.R., Sullivan K.R., Patel A.V. (2022). Association of Socioeconomic and Geographic Factors With Diet Quality in US Adults. JAMA Netw. Open.

[b0155] Monsivais P., Aggarwal A., Drewnowski A. (2014). Time Spent on Home Food Preparation and Indicators of Healthy Eating. Am. J. Prev. Med..

[b0160] Moores C.J., Bell L.K., Buckingham M.J., Dickinson K.M. (2021). Are meal kits health promoting? Nutritional analysis of meals from an Australian meal kit service. Health Promot. Int..

[b0165] National Cancer Institute. Fruit & Vegetable Screeners in the Eating at America's Table Study (EATS): Instruments By-Meal Screener https://epi.grants.cancer.gov/diet/screeners/fruitveg/instrument.html: National Institutes of Health, National Cancer Instititute, Division of Cancer Control & Population Sciences,; [updated December 14, 2021. Available from: https://epi.grants.cancer.gov/diet/screeners/fruitveg/instrument.html.

[b0170] O’Doherty Jensen K., Holm L. (1999). Preferences, quantities and concerns: socio-cultural perspectives on the gendered consumption of foods. Eur. J. Clin. Nutr..

[b0175] Pollard J., Kirk S.F.L., Cade J.E. (2002). Factors affecting food choice in relation to fruit and vegetable intake: a review. Nutr. Res. Rev..

[b0180] Robinson-Oghogho J.N., Thorpe R.J., Neff R.A. (2022). Dietary Behaviors among New Users of Meal-Kit Services during the Early Months of the COVID-19 Pandemic. Nutrients.

[b0185] Rogus S. (2018). Examining the influence of perceived and objective time constraints on the quality of household food purchases. Appetite.

[b0190] Rothgerber H. (2013). Real men don’t eat (vegetable) quiche: Masculinity and the justification of meat consumption. Psychol. Men Masculinity.

[b0195] SouthEats LLC. SouthEats About https://www.southeatscoop.com2019 [Available from: https://www.southeatscoop.com.

[b0200] StataCorp. (2017).

[b0205] Statista. Meal kits in the US: Statista Dossier on Online Meal Kit Delivery Services in the United States. https://www.statista.com/study/41244/online-meal-kit-delivery-services-in-the-us/. Published 2022. Accessed on 7/10/2022.

[b0210] Strazdins L., Griffin A.L., Broom D.H., Banwell C., Korda R., Dixon J., Paolucci F., Glover J. (2011). Time scarcity: another health inequality?. Environ. Plann. A Econ. Space.

[b0215] Sweeney L.H., Carman K., Varela E.G., House L.A., Shelnutt K.P. (2021). Shopping, and Eating Behaviors of African American and Hispanic Families: Implications for a Culturally Appropriate Meal Kit Intervention. Int. J. Environ. Res. Public Health.

[b0220] Thompson F.E., Subar A.F., Smith A.F., Midthune DOUGLAS, Radimer K.L., Kahle L.L., Kipnis VICTOR (2002). Fruit and vegetable assessment: performance of 2 new short instruments and a food frequency questionnaire. J. Am. Diet. Assoc..

[b0225] Todd JE, Mancino L, Lin B-H. The Impact of Food Away from Home on Adult Diet Quality. United States Department of Agriculture, Economic Research Service; 2010 Feb.

[b0230] Tolbert J, Kates J, Levitt L. Lifting Social Distancing Measures in America: State Actions & Metrics2020 June 15, 2022 ]. Available from: https://www.kff.org/policy-watch/lifting-social-distancing-measures-in-america-state-actions-metrics/.

[b0235] U.S. Census Bureau. QuickFacts District of Columbia United States, Population Estimates July 1, 2021 2017-2021 [Available from: https://www.census.gov/quickfacts/fact/table/DC,US/PST045221.

[b0240] Food Access Research Atlas [Internet]. U.S. Department of Agriculture (USDA). 2022 [cited June 28, 20022]. Available from: https://www.ers.usda.gov/data-products/food-access-research-atlas/.

[b0245] U.S. Census Bureau. 2021: ACS 5-Year Estimates Subject Tables: S1901 Income in the Past 12 Months [Available from: https://data.census.gov/table?t=Income+and+Earnings&g=1400000US11001009601,11001009602,11001009604.

[b0250] United States Postal Service. Every Door Direct Mail Online Tool https://eddm.usps.com/eddm/select-routes.htm?_gl=1*1u858*_ga*MTEyMDM2NDU4Ni4xNjU2NDM1MzU3*_ga_3NXP3C8S9V*MTY1NjQzNTM3Ni4xLjEuMTY1NjQzNTU5MS4w2021.

[b0255] Vadiveloo M., Perraud E., Parker H.W., Juul F., Parekh N. (2019). Geographic Differences in the Dietary Quality of Food Purchases among Participants in the Nationally Representative Food Acquisition and Purchase Survey (FoodAPS). Nutrients.

[b0260] Wark P.A., Hardie L.J., Frost G.S., Alwan N.A., Carter M., Elliott P., Ford H.E., Hancock N., Morris M.A., Mulla U.Z., Noorwali E.A., Petropoulou K., Murphy D., Potter G.D.M., Riboli E., Greenwood D.C., Cade J.E. (2018). Validity of an online 24-h recall tool (myfood24) for dietary assessment in population studies: comparison with biomarkers and standard interviews. BMC Med..

[b0265] Wolfson J.A., Smith K.C., Frattaroli S., Bleich S.N. (2016). Public perceptions of cooking and the implications for cooking behaviour in the USA. Public Health Nutr..

[b0270] Wolfson J.A., Bleich S.N., Smith K.C., Frattaroli S. (2016). What does cooking mean to you?: Perceptions of cooking and factors related to cooking behavior. Appetite.

[b0275] Zeldman J, Mialki K, Sweeney L, Shelnutt K. O27 Family Mealtime Behaviors Among Low-Income African Americans Participating in a Healthy Meal Kit Intervention. Journal of Nutrition Education and Behavior. 2020;52(7, Supplement):S13.

